# Selenium supplementation and pregnancy outcomes

**DOI:** 10.3389/fnut.2022.1011850

**Published:** 2022-10-31

**Authors:** Carl R. Dahlen, Lawrence P. Reynolds, Joel S. Caton

**Affiliations:** Center for Nutrition and Pregnancy, Department of Animal Sciences, North Dakota State University, Fargo, ND, United States

**Keywords:** selenium, supplementation, pregnancy, ovary, testis, fetus, offspring, developmental programming

## Abstract

In vertebrates and invertebrates, selenium (Se) is an essential micronutrient, and Se deficiency or excess is associated with gonadal insufficiency and gamete dysfunction in both males and females, leading to implantation failure, altered embryonic development and, ultimately, infertility. During pregnancy, Se excess or deficiency is associated with miscarriage, pre-eclampsia (hypertension of pregnancy), gestational diabetes, fetal growth restriction and preterm birth. None of this is surprising, as Se is present in high concentrations in the ovary and testes, and work in animal models has shown that addition of Se to culture media improves embryo development and survival *in vitro* in association with reduced reactive oxygen species and less DNA damage. Selenium also affects uterine function and conceptus growth and gene expression, again in association with its antioxidant properties. Similarly, Se improves testicular function including sperm count, morphology and motility, and fertility. In animal models, supplementation of Se in the maternal diet during early pregnancy improves fetal substrate supply and alters fetal somatic and organ growth. Supplementation of Se throughout pregnancy in cows and sheep that are receiving an inadequate or excess dietary intake affected maternal whole-body and organ growth and vascular development, and also affected expression of angiogenic factors in maternal and fetal organs. Supplemental Se throughout pregnancy also affected placental growth, which may partly explain its effects on fetal growth and development, and also affected mammary gland development, colostrum yield and composition as well as postnatal development of the offspring. In conclusion, Se supplementation in nutritionally compromised pregnancies can potentially improve fertility and pregnancy outcomes, and thereby improve postnatal growth and development. Future research efforts should examine in more detail and more species the potential benefits of Se supplementation to reproductive processes in mammals.

## Introduction

In both vertebrates and invertebrates, selenium (Se) deficiency or excess is associated with infertility (that is, the inability to conceive and establish a pregnancy), as reflected by small, poorly developed and poorly functioning gonads, primarily ovarian follicles in females and testes and spermatozoa in males. At least a portion of this problem is associated with implantation failure due to poor embryonic development and altered endometrial (uterine) function. Deficiency or excess of Se also is associated with reduced libido. Lastly, in terms of pregnancy, Se excess or deficiency is associated with spontaneous abortion (miscarriage), pre-eclampsia (hypertension of pregnancy), gestational diabetes, fetal growth restriction, and preterm birth (1–4).

Consistent with its known functions in cellular metabolism, in reproductive tissues Se appears to function primarily as a component of selenoproteins/selenoenzymes in a variety of antioxidant systems, including glutathione peroxidases (GPX), iodothyronine deiodinases (DIO), and thioredoxin reductases (TXNR). These major familes of antioxidant enzymes contribute to reductions in tissue reactive oxygen species and therefore minimize DNA damage (5).

## Selenium in the female reproductive tract

**FIGURE 1:**
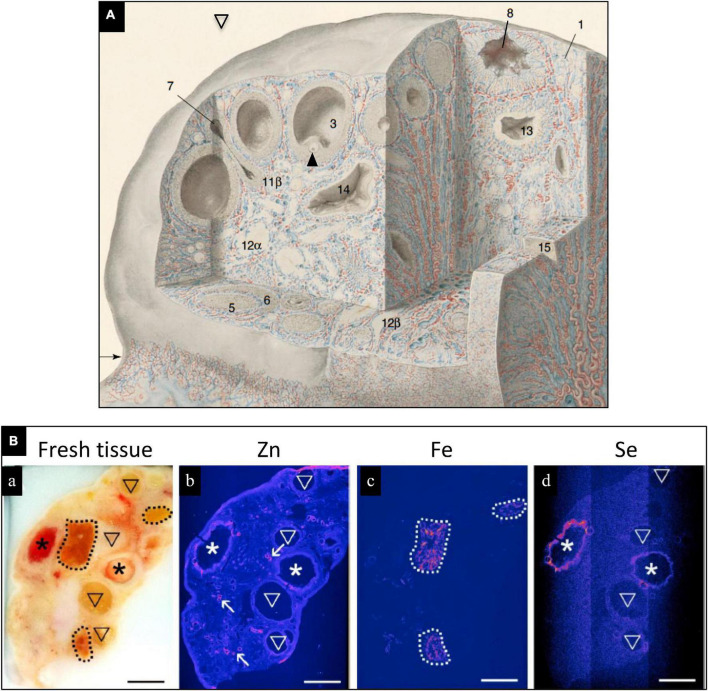
**(A)** Drawing of human ovary with sections removed to reveal histological details of an antral (preovulatory) follicle (3) containing an oocyte (arrowhead) and a postovulatory follicle that has released its oocyte and partially collapsed (8). Blood vessels are colored red (arteries) and blue (veins); modified, with permission, from Clark, 1900. **(B)** Trace elements localized in bovine ovaries by synchrotron x-ray fluorescence (S-XRF). (a) Represents fresh tissue. Zinc (b, pink) localized primarily to blood vessels, Fe (c, pink) localized primarily to corpora lutea, and Se (d, pink) localized to healthy, preovulatory follicles (*) but not to atretic (regressing) antral follicles. Modified, with permission, from Ceko et al. (6).

The bovine ovary contains high levels of Se (Figure 1), which are localized to healthy preovulatory follicles, but not in atretic follicles (6). Localization of Se in healthy pre-antral follicles indicates that Se is in close contact with the pre-ovulatory oocyte, which may play a preparatory role for subsequent fertilization, embryo development, and postnatal life. In humans, cases of low plasma, follicular fluid, amniotic fluid or tissue Se concentrations and(or) low tissue GPX concentrations or activity are associated with unexplained infertility, miscarriage, preterm birth, gestational diabetes mellitus, and small for gestational age (SGA) fetuses/newborns (1, 2, 7–9). Elevated serum levels of Se-binding protein 1, an autoantibody produced by the ovary, has been reported in women with unexplained intertility and premature ovarian failure (1, 10). In women with gestational diabetes mellitus, serum Se levels were low, and Se supplementation improved glycemic status and lipid profiles (11, 12).

After ovulation the oocyte moves to the oviduct, where fertilization and early embryo development take place. Oviductal fluid is secreted by the oviduct and acts as an embryotropic culture media for the oocyte and early embryo for their time in residence (13, 14). Addition of Se to *in vitro* fertilization cultures in animal models (cattle, dogs, pigs, yak, etc.) has resulted in positive impacts on embryo development and survival, reduced reactive oxygen species, and reduced DNA damage (15–19). Interestingly, Se-dependent mechanisms are in place to control embryo metabolic reprogramming in pro-inflammatory environments (20).

Upon deposition of semen into the reproductive tract a post-mating inflammatory response is elicited (21), and an LPS challenge of cultured bovine endometrial cells demonstrated a protective role of Se (22). *In vivo* effects of Se were demonstrated in cattle, where females receiving an organically bound source of Se had greater conceptus length compared with females receiving an inorganic source of Se (23). In addition, cattle receiving organic Se had differential expression of genes related to maternal recognition of pregnancy, including interferon-stimulated genes and progesterone-stimulated genes (23).

## Selenium in the male reproductive tract

**FIGURE 2:**
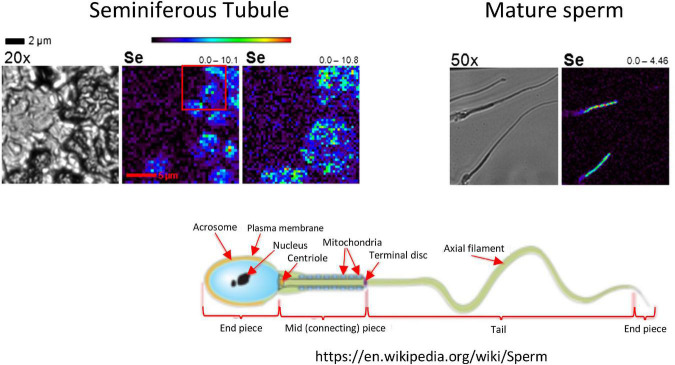
Localization of Se in mouse testis by x-ray fluorescence microscopy (XFM). In the left-hand images, a seminiferous tubule is visualized in cross section at 20×. Se (bright blue to green) localizes to elongating spermatids. In the right-hand images, Se in mature sperm localizes to the mid-piece, which is the location of sperm mitochondria (see the schematic of a mature sperm, lower right). The range below the seminiferous tubule image indicates the low and high concentrations of Se in ng/cm2. Modified, with permission, from Kehr et al. (24).

The testis contains high concentrations of Se (Figure 2), where Se has effects both in the seminiferous tubule where sperm are being produced, and in the interstitial space where testosterone production occurs and the blood supply resides (4). As sperm mature Se is localized in the mid-piece, which is also the location of sperm mitochondria (24). The action of Se is primarily as GPX4, which protects sperm from oxidative damage to their cell membranes and DNA. However, there also appears to be a specialized testes- specific isoform of TXNR (5, 25), which supports the importance of Se-containing antioxidant enzymes to testicular function and health.

In addition, greater dietary intake of Se has been associated greater sperm concentrations in semen of men infertile men (26) and some Se supplementation studies in infertile men show improvements in testicular antioxidant activity, semen Se concentrations, sperm count, sperm morphology and motility, and fertility (1–3). Selenoproteins are abundant in the testis and epididymis, include GPX4 (testis, intracellular membranes), sperm nucleus GPX4 (snGPX4), mitochondrial GPX4 (mGPX4; sperm midpiece – see Figure 2), cytosolic GPX4 (cGPX4; testis and epididymal epithelium), secreted GPX5 (epididymal lumen), cytosolic GPX3 and GPX1 (epididymal epithelium) (3). In addition, gene knockouts of selenoproteins in male mice, including mGPX4, SELENOP, snGPX4, GPX5 and global GPX4 (mGPX4, snGPX4, and cGPX4), lead to sperm abnormalities, defects in chromatin condensation in sperm, early embryonic death, and(or) increased number of miscarriages, developmental defects and neonatal mortality (3).

In terms of our understanding of the underlying mechanisms, Se excess or deficiency affects the concentrations or activities of various selenoproteins, resulting in:

• Oxidative stress/DNA damage from reactive oxygen species;
• Lack of structural integrity of sperm, affecting sperm motility and fertilization capacity;
• Defects in transport of Se into tissues, particularly testis and brain;
• Alterations in other Se effects/functions – e.g., altered gonadal morphology/size, endocrine function (e.g., thyroid), immune function, cardiovascular function, synergism with Vit E, etc. (27).

## Selenium supplementation during pregnancy

There are geographic locations and times of the year when forages grazed by livestock have insufficient Se to meet requirements. In addition, producer decisions about whether to provide supplemental mineral to grazing livestock vary widely. Therefore, our research group implemented a bovine model comparing unsupplemented beef heifers to those receiving a Se-containing mineral supplement (VTM) to understand the impacts of early gestation supplementation on maternal and fetal outcomes (28–33). An important aspect of these studies is that the control, unsupplemented heifers were receiving a basal diet that was either inadequate or in excess of requirements, both of which typically result in reduced birth weights of the offspring. Thus, they were also receiving inadequate or excess micronutrient intakes.

Evaluation of maternal and fetal samples collected on d 83 of gestation revealed heavier livers in fetuses exposed to VTM during gestation (29), and that concentrations of Se in maternal liver, fetal liver, muscle, and allantoic fluid were all greater in heifers receiving the VTM supplement. In addition, concentrations of Se in maternal liver were correlated with concentrations in fetal liver (*r* = 0.60), fetal muscle (*r* = 0.40), and allantoic fluid [*r* = 0.34; (33)]. Though no differential expression of selenoprotein transcripts was observed in the fetal or maternal portions of the placenta, VTM supplementation influenced genes related to amino acid activation, fat cell differentiation and metabolic processes (32). Amino acids are critical fuels for fetal growth and development (34) and our evaluation revealed that total amino acids and concentrations of 12 of 14 neutral amino acids evaluated in allantoic fluid were greater in heifers receiving VTM (28). Taken together, our results demonstrate that providing a Se-containing supplement during early gestation resulted in major alterations in substrate supply and(or) utilization in the fetus, indicating that research evaluating post-natal effects on health, growth, and metabolism is necessary.

In a series of studies we targeted feeding “supranutritional” (meaning above adequate but below toxic) levels of Se to pregnant ewes, fed as Se-enriched yeast or Se-enriched wheat (35–48). Again, the control, unsupplemented animals were receiving a basal diet that was either inadequate or in excess of requirements, both of which typically result in reduced birth weights of the offspring.

When fed during early pregnancy (from 21 days before until 64 days after breeding – i.e., 0.44 of pregnancy), supranutritional Se increased maternal lung mass, liver mass, and total visceral organ mass, as well as cellularity, cell proliferation and vascularity of maternal small intestine. All of these effects on the maternal system would increase metabolic capacity to support the metabolic demands of pregnancy.

Supranutritional levels of Se in the maternal diet during early pregnancy also increased fetal body mass, heart mass, lung mass, spleen mass, total visceral organ mass and large intestinal mass, as well as cell density of fetal skeletal muscle. These effects of Se supplementation would potentially improve survival and growth of the fetus and offspring. In addition, the effect on fetal skeletal muscle also has important implications for postnatal growth and carcass quality, considering that the number of myocytes in skeletal muscle is “fixed” at birth (49).

When fed throughout pregnancy, supranutritional levels of Se in the maternal diet also affected maternal whole-body and organ growth and vascular development, and these effects depended on the plane of nutrition (adequate or restricted intake). For example, Se supplementation increased maternal mammary gland vascularity at 24 h postpartum, Selenium supplementation also increased fetal body weight as well as fetal heart, lung, spleen, total visceral and large intestine weights and fetal muscle DNA concentations at 0.9 of gestation. Along with the effects on vascular development, supplemental Se throughout pregnancy also increased maternal and fetal organ expression of mRNA for vascular growth (angiogenic) factors, including NOS3 and VEGF.

Supranutritional Se fed to ewes throughout gestation also increased cell density and cell proliferation in the placenta in late pregnancy as well as lamb birth weights. As the placenta is the only source of exchange of nutrients, respiratory gases and metabolic wastes between the fetal and maternal systems (50–52), the effects of Se on placental development may explain, at least in part, the effects of supranutritional Se on fetal growth and development.

Alternatively, epigenetic mechanisms within developing offspring may also explain developmental programming responses resulting from dietary Se supplementation (53). Specifically, enzymes associated with one-carbon metabolism have been shown to be affected by Se (54, 55), while others (56) reported that Se regulates microRNAs (56) and DNA methylation (55, 57). In humans experiencing Kashin-Beck disease (associated with Se deficiencies), differentially methylated genes were reported (53). Research exploring the potential role of Se induced epigenetic changes in offspring within a developmental programming paradigm are needed to further understand the mechanisms and roles of supplemental dietary Se in developmental programming events in livestock.

Lastly, supranutritional Se fed to ewes throughout gestation also increased colostrum yield, altered colostrum composition, and increased mammary gland vascular development, and resulted in increased average daily weight gain, efficiency of growth, visceral adiposity and small intestinal mass and vascular development of the lambs postnatally. These observations further suggest a role for supranutritional supplementation of Se to the dams on developmental programming of the offspring and support the need for additional research in this area.

## Conclusion

As we have discussed, Se plays an important role in reproductive processes. Recent research with Se supplementation of sheep during nutritionally compromised pregnancies has suggested that “supranutritional” levels in the diet can positively impact pregnancy outcomes. However, these studies need to be replicated in other mammals as well. In addition, the effects of Se supplementation on other reproductive processes such as follicular development, oocyte and sperm development and maturation, fertilization and implantation, early embryonic development, and, especially, developmental programming of offspring, warrant further research as well (4, 58).

Importantly, when supranutritional maternal Se was fed as sodium selenate at 20 or 100×, or as Se-enriched wheat at 20×, of so-called “adequate” levels from day 50 to 134 (0.34–0.92) of pregnancy in ewes, no signs of selenosis were observed. These studies using sheep models of pregnancy therefore indicate that in addition to the role of dietary Se in other reproductive processes, supranutritional levels of Se fed to ewes during the periconceptual period or throughout pregnancy are not only non-toxic but can improve maternal and fetal pregnancy outcomes and postnatal growth and development. Taken together, these observations suggest to us that further research on adding Se to the diet during pregnancy is warranted in other mammals as well.

## Author contributions

All authors contributed equally to the preparation of this manuscript.
